# OHIP-14 Scores in Patients with Sjögren's Syndrome Compared to Sicca Syndrome: A Systematic Review with Meta-Analysis

**DOI:** 10.1155/2024/9277636

**Published:** 2024-05-09

**Authors:** Rossana Izzetti, Elisabetta Carli, Stefano Gennai, Filippo Graziani, Marco Nisi

**Affiliations:** ^1^Department of Surgical, Medical and Molecular Pathology and Critical Care Medicine, University of Pisa, Pisa, Italy; ^2^Unit of Dentistry and Oral Surgery, University Hospital of Pisa, Pisa, Italy

## Abstract

**Objectives:**

Primary Sjögren's syndrome (pSS) is a chronic autoimmune disorder characterized by dryness of mucous membranes, predominantly the eyes and mouth, following glandular tissue substitution. The onset of oral dryness constitutes a significant source of discomfort that negatively affects overall quality of life. This systematic review aimed at investigating the differences in Oral Health Impact Profile-14 (OHIP-14) questionnaire scores in patients diagnosed with Sjögren's syndrome compared to sicca syndrome, to assess the influence of the two conditions on oral health. *Study Design*. A systematic electronic and manual search was performed up to December 2023 for studies reporting OHIP-14 questionnaire scores in pSS patients versus sicca syndrome. Two authors independently reviewed, selected, and extracted the data. The outcome was the assessment of OHIP-14 scores in studies comparing pSS- and sicca syndrome-affected patients. Meta-analysis was conducted on available quantitative data.

**Results:**

Literature search retrieved 30 articles, and 3 articles met the criteria for inclusion in the review. Meta-analysis revealed significantly higher scores in patients with sicca syndrome compared to pSS, although salivary flow was markedly reduced in pSS.

**Conclusions:**

While offering supplementary information to standard tests and supporting the assessment of pSS and sicca syndrome patients, further validation is necessary to establish OHIP-14 validity in determining the impact of pSS and sicca syndrome on patients' quality of life.

## 1. Introduction

Primary Sjögren's syndrome (pSS) is a complex systemic autoimmune disorder characterized by the dysfunction of exocrine salivary and lachrymal glands following progressive chronic lymphocytic infiltration in the glandular parenchyma [1]. The diagnosis involves serology, histopathology, and functional tests to detect signs of disease. Being the clinical presentation extremely variable and the laboratory markers employed nonspecific, the diagnostic work-up is often quite complex.

Over the years, several attempts to frame pSS through the application of diagnostic criteria have been made, resulting in the American–European Consensus Group (AECG) criteria and in the American College of Rheumatology and the European League Against Rheumatism (ACR/EULAR) criteria [1, 2]. The 2002 AECG criteria established six items, namely, ocular symptoms, oral symptoms, ocular signs, histopathology, salivary glands involvement, and antibodies. The positivity of at least four of the AECG items including histology and antibodies was deemed diagnostic of pSS and allowed to stratify the presence of secondary SS [1]. The 2016ACR/EULAR classification criteria validated the application of five items of different weights for the diagnosis of pSS [2]. In particular, a positive histology of minor salivary glands with a focus score ≥1.3 and the presence of positive anti-SSA (Ro) antibodies were assigned a score of 3, while the remaining items (ocular staining score ≥5 or van Bijsterveld score ≥4 on at least one eye, Schirmer ≤5 mm/5 min on at least one eye, and unstimulated whole saliva flow rate ≤0.1 ml/min) were assigned one point each. A total score ≥4 indicated the presence of pSS [2]. Interestingly, these criteria should be applied in patients who do not present potential confounding conditions accountable for the development of xerostomia, including history of head and neck radiation treatment, active hepatitis C virus infection, acquired immunodeficiency syndrome, sarcoidosis, amyloidosis, graft versus host disease, or IgG4-related disease [2].

Sicca syndrome, also known as sicca complex, is a chronic medical condition characterized by the pervasive symptom of dryness, particularly affecting mucous membranes of the eyes, mouth, and other moisture-producing areas [1]. While sicca syndrome shares similarities with pSS, it is important to note that individuals with sicca may not meet the complete diagnostic criteria for Sjögren's syndrome. Indeed, despite encompassing the same cohort of symptoms as pSS, the absence of focal sialadenitis or positive antibodies is noted [1]. Interestingly, in both pSS- and sicca syndrome-affected patients, xerophthalmia and xerostomia represent the major complaints, being the initial and most disabling symptoms related to an overall worsening of patients' quality of life [3].

Oral health plays a crucial role in the well-being of individuals, and its impact can be particularly significant in patients suffering from autoimmune conditions affecting salivary glands due to the reduction in salivary flow [4]. Oral Health Impact Profile (OHIP-14) is a 14-item questionnaire investigating the impact of various aspects of oral health assessed through seven domains, specifically functional limitations, physical pain, psychological discomfort, physical disability, social disability, and handicap [5, 6]. Higher OHIP-14 scores indicate worse self-perception of quality of life and an overall lower oral health-related quality of life (OHRQoL) [7].

This systematic review aimed to explore potential disparities in OHIP-14 scores and salivary flow between patients diagnosed with Sjögren's syndrome and patients with sicca syndrome to elucidate the distinct impacts of these two conditions on oral health.

## 2. Materials and Methods

### 2.1. Study Protocol

The protocol for this study was developed according to the Preferred Reporting Items Systematic Review and Meta-Analyses Extension Statement for Reporting of Systematic Reviews Incorporating Network Meta-Analyses of Health Care Interventions [8–10]. The protocol was registered at the International Prospective Register of Systematic Reviews (PROSPERO; registration number CRD42023447371).

The following focused question was phrased:

“What is the impact of pSS and sicca syndrome on oral health assessed through OHIP-14?”

The following PECOS was employed for study inclusion:

(P) Population: adult patients.
(E) Exposure: patients with a diagnosis of pSS.
(C) Comparison: patients affected by sicca syndrome.
(O) Type of outcome measures: OHIP-14 score and salivary flow.
(S) Type of study: observational case–control studies and cross-sectional studies.

Interventional studies were excluded as changes in OHIP-14 scores following treatment were not in the scope of the present review. Review articles and systematic reviews were not included, although the bibliographies were screened to search for potentially relevant articles. No time limitations were set. Only articles in English were included.

### 2.2. Information Sources and Search

An electronic literature search was performed in the Cochrane Oral Health Group specialist trials, MEDLINE via PubMed, and EMBASE up to June 2023 using a combination of MeSH terms and free text words ((“Sjögren's Syndrome”(Mesh) OR “Sjögrens Syndrome” OR “Syndrome, Sjögren's” OR “Sjögren Syndrome”) AND (“Oral Health”(Mesh) OR “Oral Health Related Quality of Life” OR “Oral Health Impact Profile” OR “OHIP-14”)).

The search strategy was first designed for the MEDLINE database and was then modified for the other databases. Trial databases such as clinicaltrial.gov and other relevant sites were searched. Manual search was also performed, and bibliographies of all relevant papers and review articles were checked to detect additional studies.

### 2.3. Study Selection and Data Collection

Two calibrated reviewers (RI and MN) screened the articles for possible inclusion in the review. Reviewers underwent calibration for study screening by comparing their assessments to those of another reviewer experienced in conducting systematic reviews (SG). Each round of calibration involved independently assessing the validity of 20 titles and abstracts from the search in duplicate, until a consistent level of agreement measured by Cohen's *κ*-score was attained. Title and abstract analysis was performed to screen the articles retrieved from the literature search. In cases of unclear abstracts, full-text analysis was performed not to exclude any potentially relevant articles. Data from the articles included following full-text analysis were extracted and synthesized.

### 2.4. Risk of Bias

The quality assessment and the risk of bias of the included studies were performed according to the ROBINS-E tool (Risk Of Bias In Non-randomized Studies—of Exposure) [11, 12] by two independent reviewers (RI and MN). In cases of critical or serious judgment, the study was considered at high risk of bias. Tables were generated using the Robvis tool [13].

### 2.5. Statistical Analysis

Data were expressed through evidence tables reporting on study characteristics and conclusions. OHIP-14 scores were reported as the mean and standard deviation (SD).

Meta-analysis was performed using the random effects model and presented through graphs and forest plots using OpenMeta [Analyst] (http://www.cebm.brown.edu/openmeta/).

OHIP-14 scores, expressed as the weighted mean difference (WMD), were compared in pSS and sicca syndrome patients. A 95% confidence interval (CI) was set for continuous outcomes. The analysis was carried out using the standardized mean difference as the outcome measure. Tau^2^ was employed to assess heterogeneity using the restricted maximum-likelihood estimator [14]. Studentized residuals and Cook's distances were employed to detect potential outliers and/or overly influential studies. Funnel plot asymmetry was checked with rank correlation and regression tests, using the standard error of the observed outcomes as a predictor.

## 3. Results

### 3.1. Study Selection

**Figure 1:**
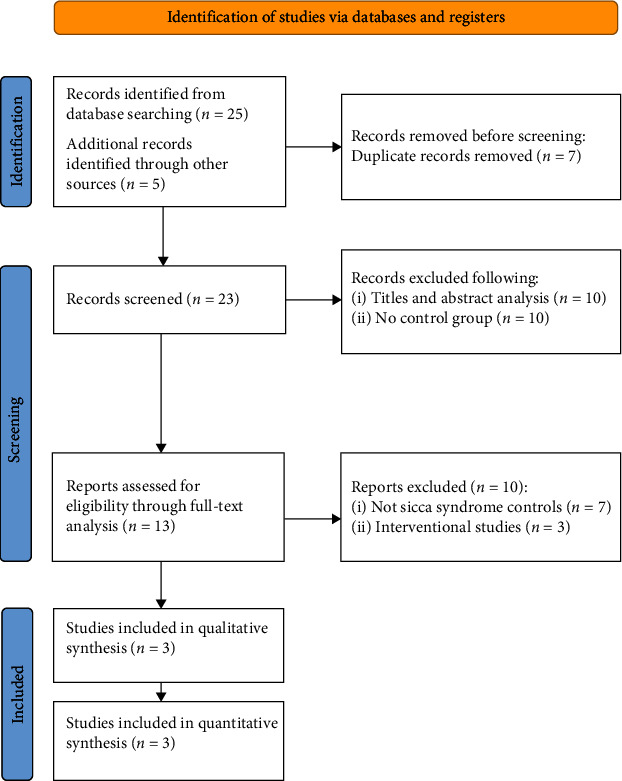
PRISMA flowchart (PRISMA 2020 flow diagram for new systematic reviews which included searches of databases and registers. Source: Page MJ, McKenzie JE, Bossuyt PM, BoutronI, Hoffmann TC, Mulrow CD, et al. The PRISMA 2020 statement: an updated guideline for reporting systematic reviews. BMJ 2021;372:n71.doi:10.1136/bmj.n71).

In total, 25 studies were retrieved from literature search. Manual search further retrieved five articles. After the removal of duplicates, title and abstract analysis was performed on 23 articles, and 13 studies were selected for full-text analysis (see Table 1 for the reasons for article exclusion). Further exclusion of 10 articles led to the performance of the final assessment on three articles (Figure 1). A *κ*-score > 0.8 was obtained among the reviewers in all the phases of title and abstract selection, full-text analysis, and data extraction.

### 3.2. Population Characteristics

The study population consisted of 133 pSS patients (128 females), with a mean age of 52.7 years (SD 17.1), and 54 sicca syndrome patients (49 females, mean age 49.4 ± 15.9 years). The mean OHIP-14 score was 15.3 (SD 12.9) in pSS patients and 17.9 (SD 9.7) in sicca syndrome patients. The studies [35–37] included in the systematic review are summarized in Table 2.

### 3.3. Outcomes Reported in the Included Studies

Azuma et al. [35] evaluated the correlation between salivary secretion and quality and oral health assessed through OHIP-14. The authors reported a reduced salivary flow in pSS patients compared to controls associated with higher OHIP-14 scores.

Tashbayev et al. [36] reported lower OHIP-14 scores for pSS patients compared to sicca syndrome patients. Although individuals with sicca syndrome exhibited milder clinical manifestations compared to pSS patients, their overall quality of life was notably inferior.

Galves et al. [37] found higher OHIP-14 scores in patients affected by sicca compared to pSS. An overall poorer oral health was noted in pSS patients related to reduced salivary flow, dental, and periodontal problems.

### 3.4. Meta-Analysis of OHIP-14 Scores

**Figure 2:**
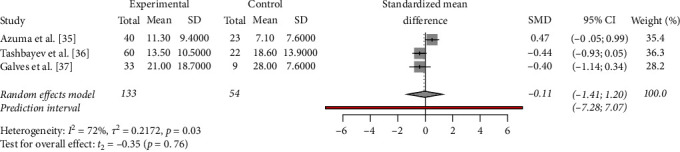
Meta-analysis of the included studies.

The meta-analysis of OHIP-14 scores revealed significant heterogeneity among the included studies, with *I*^2^ = 72% (*p*=0.03). The estimated between-study variance (*t*^2^) was 0.2172, suggesting considerable dispersion in effect sizes. Despite this variability, the overall effect size, represented by the standardized mean difference (SMD), was found to be −0.11 with a 95% confidence interval of (−1.41 to 1.2), indicating a small but statistically significant difference in OHIP-14 scores using a random effects model. The corresponding prediction interval (−7.28; 7.07) suggested that the true effect size was likely to fall within this range with 95% confidence. Notably, the *p*-value of 0.03 further supports the significance of the observed effect (Figure 2).

**Figure 3:**
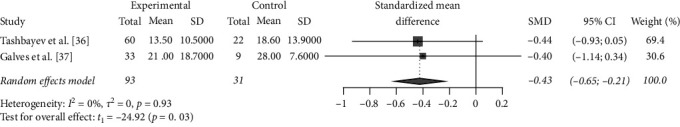
Meta-analysis result after the exclusion of the study by Azuma et al. [35].

Since only the study by Azuma et al. [35] reported higher scores for pSS compared to sicca patients, an additional numerical synthesis of the results was performed, with the exclusion of the aforementioned study. Based on the analysis performed using the random effects model with an inverse variance method, a statistical difference was noted between the two cohorts, with a summarized SMD of −0.43 with a 95% confidence interval (−0.65 to −0.21) (Figure 3).

### 3.5. Meta-Analysis of the Differences in Salivary Flow

The meta-analysis of salivary flow yielded significant heterogeneity, as evidenced by an *I*^2^ value of 88%. This indicates substantial variability in effect sizes among the included studies. The estimated between-study variance (*t*^2^) was 0.6976, suggesting considerable dispersion in salivary flow measurements across studies. The *p*-value of less than 0.01 indicated that the observed heterogeneity is statistically significant.

**Figure 4:**
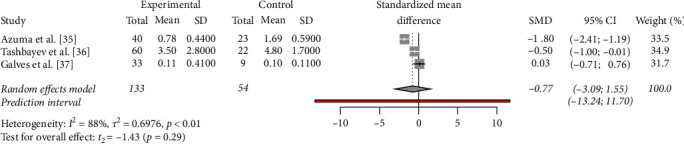
Meta-analysis of the differences in salivary flow.

The SMD in salivary flow between pSS patients and those with sicca syndrome was calculated to be −0.77. This negative SMD suggested that, on average, salivary flow is lower in patients with pSS compared to those with sicca syndrome. However, it is important to note that the confidence interval for this estimate (−3.09 to 1.55) is wide and includes zero, indicating uncertainty in the true effect size. This suggests that the difference in salivary flow between the two groups may not be statistically significant (Figure 4).

### 3.6. Risk of Bias

**Figure 5:**
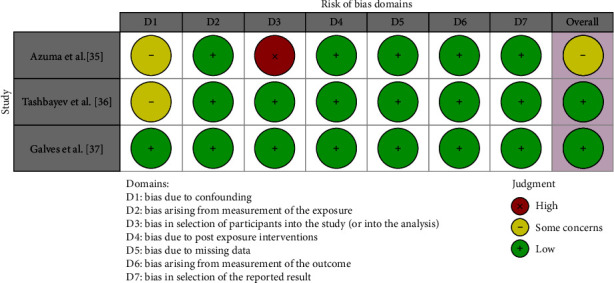
Risk of bias assessment with the ROBINS-E tool.

In the study by Azuma et al. [35], some concerns were raised in domains D1 and D3, indicating potential issues related to selection of participants which was deemed critical in association with a poor management of confounding factors, while the remaining domains showed a low risk of bias. The study by Tashbayev et al. [36] demonstrated low risk across all domains, suggesting a generally robust study design and conduct. Similarly, the study by Galves et al. [37] exhibited consistently low risk across all domains, indicating a high level of methodological quality. Overall, one study [35] showed a moderate risk of bias, while two studies [36, 37] were deemed at low risk of bias (Figure 5). None of the studies was judged at high risk of bias.

## 4. Discussion

The present review highlights the presence of oral health impairment in both pSS and sicca syndrome patients. However, it appears noteworthy that OHIP-14 scores were higher in patients affected by sicca syndrome compared to pSS. This result is consistent with current literature, which reports a paucity of evidence on the actual impact of pSS and sicca syndrome on oral health and an overall difficulty in the assessment of oral symptoms burden in the presence versus absence of disease. The analysis of salivary flow indicated a trend towards lower salivary flow in patients with pSS compared to those with sicca syndrome, although the substantial heterogeneity and wide confidence intervals highlight the need for cautious interpretation of the results. Further research with larger sample sizes and more standardized methodologies is warranted to better understand the differences in salivary flow between these patient groups.

Indeed, the exact reasons for higher OHIP-14 scores in sicca patients compared to pSS may vary and could be influenced by several factors. Sicca syndrome encompasses a broader spectrum of conditions characterized by dry mouth and dry eyes, including but not limited to Sjögren's syndrome [1]. Some sicca patients may experience more severe symptoms than those with Sjögren's syndrome alone, leading to greater oral health-related impact and thus higher OHIP-14 scores [37]. While both Sjögren's syndrome and sicca syndrome involve salivary gland dysfunction, the underlying mechanisms may differ. Sicca syndrome can result from various factors such as medications and coexist with other conditions, including autoimmune diseases, hormonal changes, or aging, whereas Sjögren's syndrome specifically involves autoimmune reaction of the salivary glands [1]. The higher OHIP-14 scores observed in sicca patients compared to Sjögren's syndrome may stem from the complex interplay of symptom severity, underlying pathophysiology, comorbidities, treatment effects, and psychosocial factors [36, 37].

In the literature, the symptoms burden of pSS has been extensively investigated. However, the heterogeneity in disease presentation and the significant psycho-affective component of the disease often contribute to inherent challenges in interpreting the results [38]. Indeed, symptoms associated with mouth and eye dryness are the most frequently reported by the patients and predominant in terms of prevalence [39]. Previous studies involved the stratification of patients depending on symptoms burden in terms of dryness, fatigue, pain, anxiety, and depression [40, 41]. According to Tarn et al. [40], pSS patients can be classified as high symptoms burden (HSB), low symptoms burden (LSB), dryness-dominant fatigue (DDF), and pain-dominant fatigue (PDF). In the study by McCoy et al. [41], DDF and PDF groups are substituted by dryness-low pain (DLP) and dryness-high pain (DHP). The HSB group was confirmed to have a major impact on OHRQoL [40], although showing reduced organ involvement and laboratory abnormalities [41]. Sjögren's syndrome diagnosis can be complex and involves a multidisciplinary evaluation including functional tests, ultrasonography, and histology [42]. Glandular involvement can be reliably assessed through ultrasonography, although obtaining histological confirmation is of utmost importance for the diagnosis [43–46]. However, the correspondence between patient-reported outcomes and actual glandular involvement is not always observed, as several factors appear to affect the patient's perception of the disease.

Despite being a validated and comprehensive questionnaire investigating various aspects of oral conditions, including discomfort, dysfunction, and disability, the OHIP-14 questionnaire shows some limitations in the assessment of patients affected by xerostomia. It is important to note that the OHIP-14 questionnaire has not been specifically developed to assess oral dryness problems. Therefore, OHIP-14 scores could potentially be influenced by other dental or oral health complaints experienced by individuals, which may not be adequately captured by this instrument. On the one hand, it appears overall difficult to estimate the impact of sicca symptoms in patients affected by pSS versus controls. In fact, it has been reported that OHIP-14 questionnaire scores can seldom discriminate between pSS patients and controls, as similar outcomes are observed [47]. On the other hand, while xerostomia is a complex condition involving several mechanisms related to salivary glands hypofunction, the correlation between sicca symptoms and salivary flow appears still uncertain, as sicca symptoms can appear even in the presence of preserved salivary flow [48]. As highlighted by previous literature [49–51], symptoms burden and quality-of-life impairment between Sjögren's syndrome and sicca syndrome appear similar. Cho et al. [49] reported a reduction in quality of life by the same extent in both pSS and sicca patients, despite a difference between groups with regard to clinical symptoms and depression/anxiety. Such an aspect was confirmed by Chou et al. [50], who highlighted that in symptomatic patients, the presence of a pSS diagnosis corresponded to better emotional and psychological well-being compared to patients without a diagnosis. Finally, Pucino et al. [51] did not report significant differences in terms of quality of life, depression, and anxiety between pSS and sicca patients. Since dryness can occur either in patients affected by pSS or in patients with idiopathic sicca syndrome, according to current evidence, it appears that most of the symptoms could also be ascribed to other factors, including anxiety, depression, and fatigue. Anxiety and depression, however, do not seem to differ in pSS-affected patients versus sicca controls, as a weak correlation with functional tests has been observed [35]. Similarly, chemosensory function alterations, evaluated as the occurrence of olfactory and gustatory alterations, the presence of halitosis, and burning sensation, were reported to be present in both pSS and sicca syndrome in the absence of significant differences between groups, while concurring to an overall poorer OHRQoL [52]. In particular, a decline in quality of life is reported to occur quite rapidly in patients with elevate symptoms burden, thus requiring an effective therapeutic strategy [41].

Notably, recent evidence suggests that symptoms burden is not necessarily correlated with disease severity markers, such as positive serology and extraglandular involvement [42]. From this perspective, the management of this discordance between disease severity and symptoms burden requires a therapeutic approach designed on the specific disease phenotype [44].

The present study has some limitations. The meta-analysis comparing OHIP-14 scores in pSS versus sicca patients encounters several potential sources of heterogeneity that may impact the interpretation of results. First, the discrepant cohort sizes between Sjögren and sicca patients could introduce heterogeneity. Larger sample sizes in the pSS group may affect the precision of estimates and contribute to variability in OHIP-14 scores. Heterogeneity and the observed risk of bias underscore the importance of cautious interpretation of meta-analysis results. Conducting subgroup analyses based on disease severity or symptomatology, as well as sensitivity analyses to assess the impact of risk of bias, could help elucidate the sources of heterogeneity and enhance the robustness of conclusions drawn from the meta-analysis. Additionally, acknowledging the inherent variability between pSS and sicca patients and the potential impact on OHIP-14 scores is essential for interpreting the findings accurately. With only three studies meeting the eligibility criteria, there may be insufficient statistical power to detect subtle differences between pSS and sicca patients in terms of OHIP-14 scores. Additionally, the limited number of studies may exacerbate the impact of heterogeneity and bias, potentially skewing the pooled estimates and compromising the robustness of conclusions drawn from the meta-analysis. The paucity of literature on the topic and the heterogeneity of the studies, as well as the limited number of studies, may thus affect the robustness of the meta-analysis and generalizability of the findings.

To improve the assessment of quality-of-life impairment in patients with pSS and sicca syndrome, future research should be focused at improving the assessment of disease-specific symptoms, including dry mouth, dry eyes, fatigue, joint pain, and cognitive dysfunction which could be evaluated alongside oral health-related outcomes to provide a deeper understanding of patients' experiences and needs. Moreover, the interplay between disease-related factors and psychosocial variables may represent a relevant aspect impacting on quality of life. Longitudinal studies tracking patients over time could provide valuable insights into the trajectory of quality-of-life impairment in Sjögren's syndrome and sicca syndrome. By assessing changes in symptoms, treatment effects, and quality-of-life outcomes over time, the dynamics of pSS and sicca syndrome and their influence on long-term outcomes could be better understood. By implementing these recommendations, future research could enhance the assessment of quality-of-life impairment in patients with pSS and sicca syndrome, leading to improved patient care and better treatment outcomes. Nevertheless, the present review raises awareness on the difficulty of the assessment of oral symptoms burden in sicca patients.

In conclusion, an overall worsening of oral health as evaluated by means of OHIP-14 is noted in patients affected by pSS. However, an even worse impact can be observed in the presence of sicca syndrome, even though these patients lack the typical pathogenetic pattern of Sjögren's syndrome. Further studies are encouraged to improve the assessment of quality-of-life impairment in patients with pSS and sicca syndrome.

## Figures and Tables

**Table 1:** Reasons for study exclusion following title and abstract and full-text analyses.

No control group	Healthy controls	Interventional studies
Amaral et al. [15] Enger et al. [16] Fernández Castro et al. [17] Nesvold et al. [18] Rojas-Alcayaga et al. [19] Serrano et al. [20] Stewart et al. [21] Vujovic et al. [22] Yalcinkaya et al. [23] Yenissoy et al. [24]	Azuma et al. [25] Azuma et al. [26] Li et al. [27] Molania et al. [28] Moreno-Quispe et al. [29] Rusthen et al. [30] Šijan Gobeljić et al. [31]	da Mata et al. [32] López-Pintor et al. [33] Mumcu et al. [34]

**Table 2:** Synthesis of studies reporting of pSS patients versus sicca syndrome patients.

Authors	Country	Study design	Setting	Funding	Diagnostic criteria	pSS	Sicca syndrome	Conclusions
Azuma et al. [35]	Japan	Cross-sectional	University hospital	Public funding	American–European Consensus Group	40 patients (37 F) Age 55.4 years (SD 13.2) OHIP-14 11.3 (9.4) Salivary flow 0.78 ± 0.44 ml (min)	23 patients (18 F) Age 56.1 years (SD 17.4) OHIP-14 7.1 (SD 7.6) Salivary flow 1.69 ± 0.59 ml (min)	The decrease in salivary flow and salivary EGF levels associated with pSS progression causes a deterioration of saliva quality contributing to an impairment in oral health

Tashbayev et al. [36]	Norway	Cross-sectional	University hospital	Public funding	American–European Consensus Group	60 (60 F) Age 53.6 years (SD 13.2) OHIP-14 13.5 (SD 10.5) Salivary flow 3.5 ± 2.8 ml (min)	22 patients (22 F) Age 52 years (SD 10.4) OHIP-14 18.6 (SD 13.9) Salivary flow 4.8 ± 1.7 ml (min)	Even though patients affected by sicca syndrome had less severe clinical signs than the pSS patients, they demonstrated much poorer general and oral health-related quality of life

Galves et al. [37]	Brazil	Cross-sectional	University hospital	Public funding	American–European Consensus Group	33 (31 F) Age 49 years (SD 24.9) OHIP-14 21 (SD 18.7) Salivary flow 0.11 ± 0.41 ml (min)	9 patients (9 F) Age 40 years (SD 19.9) OHIP-14 28 (7.6) Salivary flow 0.10 ± 0.15 ml (min)	An association between pSS and oral health impairment was noted, with a negative impact on quality of life

## Data Availability

Data are available within the article.
